# Reduced Insulin Resistance and Oxidative Stress in a Mouse Model of Metabolic Syndrome following Twelve Weeks of Citrus Bioflavonoid Hesperidin Supplementation: A Dose–Response Study

**DOI:** 10.3390/biom14060637

**Published:** 2024-05-29

**Authors:** Abdulsatar Jamal, Holly Brettle, Dina A. Jamil, Vivian Tran, Henry Diep, Alexander Bobik, Chris van der Poel, Antony Vinh, Grant R. Drummond, Colleen J. Thomas, Maria Jelinic, Hayder A. Al-Aubaidy

**Affiliations:** 1 Centre for Cardiovascular Biology and Disease Research, La Trobe Institute for Molecular Science (LIMS), & Department of Microbiology, Anatomy, Physiology & Pharmacology (MAPP), La Trobe University, Bundoora, VIC 3086, Australia; 20382699@students.latrobe.edu.au (A.J.); h.brettle@latrobe.edu.au (H.B.); dina.jamil@newmedschool.com.au (D.A.J.); vivian.tran@latrobe.edu.au (V.T.); h.diep@latrobe.edu.au (H.D.); alex.bobik@baker.edu.au (A.B.); c.vanderpoel@latrobe.edu.au (C.v.d.P.); a.vinh@latrobe.edu.au (A.V.); g.drummond@latrobe.edu.au (G.R.D.); colleen.thomas@latrobe.edu.au (C.J.T.); 2 NewMed Education Australia, Hamilton, QLD 4007, Australia; 3 Baker Heart and Diabetes Research Institute, Melbourne, VIC 3004, Australia; 4 Australian Institute for Musculoskeletal Science, Melbourne, VIC 3021, Australia; 5 Pre-Clinical Critical Care Unit, Florey Institute of Neuroscience and Mental Health, University of Melbourne, Parkville, VIC 3052, Australia

**Keywords:** hesperidin, metabolic syndrome, HbA1c, insulin, 8-hydroxydeoxyguanosine

## Abstract

Metabolic syndrome (MetS) is a cluster of metabolic abnormalities affecting ~25% of adults and is linked to chronic diseases such as cardiovascular disease, cancer, and neurodegenerative diseases. Oxidative stress and inflammation are key drivers of MetS. Hesperidin, a citrus bioflavonoid, has demonstrated antioxidant and anti-inflammatory properties; however, its effects on MetS are not fully established. We aimed to determine the optimal dose of hesperidin required to improve oxidative stress, systemic inflammation, and glycemic control in a novel mouse model of MetS. Male 5-week-old C57BL/6 mice were fed a high-fat, high-salt, high-sugar diet (HFSS; 42% kcal fat content in food and drinking water with 0.9% saline and 10% high fructose corn syrup) for 16 weeks. After 6 weeks of HFSS, mice were randomly allocated to either the placebo group or low- (70 mg/kg/day), mid- (140 mg/kg/day), or high-dose (280 mg/kg/day) hesperidin supplementation for 12 weeks. The HFSS diet induced significant metabolic disturbances. HFSS + placebo mice gained almost twice the weight of control mice (*p* < 0.0001). Fasting blood glucose (FBG) increased by 40% (*p* < 0.0001), plasma insulin by 100% (*p* < 0.05), and HOMA-IR by 150% (*p* < 0.0004), indicating insulin resistance. Hesperidin supplementation reduced plasma insulin by 40% at 140 mg/kg/day (*p* < 0.0001) and 50% at 280 mg/kg/day (*p* < 0.005). HOMA-IR decreased by 45% at both doses (*p* < 0.0001). Plasma hesperidin levels significantly increased in all hesperidin groups (*p* < 0.0001). Oxidative stress, measured by 8-OHdG, was increased by 40% in HFSS diet mice (*p* < 0.001) and reduced by 20% with all hesperidin doses (*p* < 0.005). In conclusion, hesperidin supplementation reduced insulin resistance and oxidative stress in HFSS-fed mice, demonstrating its dose-dependent therapeutic potential in MetS.

## 1. Introduction

Metabolic syndrome (MetS) represents the largest burden of a non-communicable disease globally, with ~25% of the global population estimated to have MetS [1]. Adults with MetS are three times more likely to have a heart attack or stroke and twice as likely to die compared to those without MetS [2]. An individual is diagnosed with MetS if they have three or more of the following metabolic disturbances: increased waist circumference, increased blood glucose levels (hyperglycemia), increased blood insulin levels (hyperinsulinemia), increased triglycerides (TGs), reduced high density lipoprotein (HDL) levels, and elevated blood pressure [3].

The pathophysiology of MetS is influenced by a variety of factors, with oxidative stress playing a central role [4]. This condition arises when an imbalance between the production of reactive oxygen species (ROS) and the body’s ability to detoxify these reactive intermediates or repair the resulting damage occurs [5]. Key contributors to this oxidative stress include increased adiposity and hyperglycemia due to insulin resistance [6]. These factors not only contribute directly to oxidative stress but also lead to secondary complications such as hypertension, elevated levels of blood lipids (dyslipidemia), and inflammation. The interconnected nature of these disturbances underscores the pivotal role of oxidative stress in the progression of MetS, marking it as a critical indicator in its development. There are multiple mechanisms as to how ROS drives the pathophysiology of MetS [6]. ROS damage vital organs, such as the blood vessels, kidneys, heart, liver, pancreas, and brain, which can further exacerbate metabolic disturbances such as hypertension and hyperglycemia [7]. ROS are typically generated by decreased aerobic respiration, catecholamine oxidation, and nitric oxide (NO) production [8]. An oxidized derivative of deoxyribonucleic acid (DNA), called 8-hydroxy-2′-deoxy-guanosine (8-OHdG), is a well-established marker of oxidative stress [9]. Unlike other markers of oxidative stress, 8-OHdG is stable and easily measured in plasma [10].

Patients with MetS also display chronic inflammation, and this is thought to be a major contributor to the manifestation of its many symptoms and progression to tissue damage and disease [11]. Chronic inflammation is characterized by increased levels of proinflammatory markers such as adipokines, cytokines, and chemokines, as well as infiltration of activated leukocytes (particularly macrophages) into adipose and other tissue reservoirs. Inflammation in MetS contributes to the progression and development of insulin resistance, type 2 diabetes mellitus (T2DM), dyslipidemia, and atherosclerosis [12]. Cytokines such as leptin, adiponectin, interleukin-6 (IL-6), and tumor necrosis factor-alpha (TNF-α) are all major drivers of inflammation in MetS [13]. Additionally, C-reactive protein (CRP), produced by the liver, is an important marker for cardiovascular disease (CVD), T2DM, and MetS [14,15]. Inflammation can therefore act as a positive feedback loop, where MetS causes inflammation, causing further injury to tissues and cells, and leading to further progression of disease.

Currently, MetS therapies are largely aimed at targeting individual metabolic disturbances; however, there are only a few treatments that target several of these [16]. Moreover, many of these therapies have off-target effects. For example, statins can induce gastrointestinal disturbances, insomnia, and rashes, while gliptins can induce headaches and diarrhea [17,18]. Antihypertensives such as thiazide diuretics can promote weakness, fatigue. and headaches [19]. Weight reduction is a primary therapy for combating MetS, as it can positively impact multiple metabolic disturbances simultaneously [20]. However, achieving and maintaining significant weight loss can be challenging for many patients [20]. Many MetS patients are required to take more than one medication to treat the multiple factors of the syndrome [21]. The individual and combined side effects of these current “MetS” therapies ultimately contribute to poor compliance from patients [22]. Poor compliance, in combination with the poor efficacy of current treatments and the increasing prevalence of MetS, highlights the urgent unmet need for new and improved therapies for these patients.

Hesperidin (C_25_H_34_O_15_) is a citrus bioflavonoid (natural phytochemical), predominantly found in oranges and lemons, with an average fruit containing approximately 20–60 mg of hesperidin [23]. Hesperidin has very low toxicity and is one of the most abundant bioflavonoids found in citrus fruits, which justifies the focus on hesperidin in this study [24,25]. Separate animal studies suggest that hesperidin has anti-inflammatory, anti-hyperlipidemic, anti-hypertensive, anti-microbial, antioxidant, and immune-boosting properties [26,27,28]. In mice, doses of ~500 mg/kg did not have any negative health impacts, with a lethal dose determined to be 4.8 g/kg [29]. Following 2 weeks of a high-fat diet and streptozotocin (STZ) injections in rats, administration of 50 mg/kg hesperidin (oral) decreased inflammatory cytokines in adipose tissue [27]. Additionally, systolic blood pressure (BP) was significantly reduced by ~9 mmHg in spontaneously hypertensive rats ~12 h after hesperidin administration (50 mg/kg/day) [28]. In hesperidin-administered patients (400 mg/day for 8 weeks), total cholesterol, blood glucose levels, free fatty acids, and TG levels were reduced [30]. Collectively, these beneficial effects of hesperidin likely decrease CVD progression [31,32,33]; however, further work is needed to clarify this, particularly in the setting of MetS, where multiple metabolic disturbances are concurrently present.

Hesperidin is also hypothesized to target the DPP-4 pathway in a similar way to gliptins. Although hesperidin is not classified as a DPP-4 inhibitor, in vitro studies show high concentrations of hesperidin can inhibit the DPP-4 enzyme, making it a possible alternative to gliptin therapy [34]. The mechanism of action of hesperidin is yet to be fully understood, but hesperidin’s DPP-4 inhibition is thought to drive the improved plasma glucose levels seen in patients consuming a Mediterranean diet [35]. As part of the objectives for our study, we explore whether in vivo hesperidin treatment can inhibit DPP-4 activity, particularly in the setting of MetS, where it could potentially improve multiple metabolic disturbances like hyperglycemia and hyperinsulinemia.

Additionally, there has been no determination of the optimal dose of hesperidin to provide the most beneficial outcomes in MetS, and hence, this study tested the hypothesis that daily hesperidin supplementation over a 12-week period at varying doses (70 mg/kg, 140 mg/kg, and 280 mg/kg) would lead to dose-specific reductions in markers of inflammation and oxidative stress in mice with metabolic syndrome, as well as an improvement in glycemic control.

## 2. Materials and Methods

### 2.1. Animals

All animal studies followed the National Health and Medical Research Council (NHMRC) of Australia code of practice for the care and use of animals for research purposes. Animal studies were approved by the La Trobe University Animal Ethics Committee (AEC 21003). Male C57BL/6J mice were acquired from the AgriBio facility (La Trobe University, Bundoora, VIC, Australia) at 4–5 weeks of age. Mice were kept on a 12 h light/dark cycle with ad libitum access to food and water (except when undertaking fasting for study timeline FBG measurements). Mice were housed in Tecniplast (ventilated and bio-exclusion) cages with littermates (maximum 5 mice per cage) in the Central Animal House at La Trobe University, Bundoora.

A total of 45 mice were used in this study. Mice arrived at 5 weeks of age and were acclimatized to their new surroundings for one week prior to handling. At 6 weeks of age, baseline systolic BP, fasted blood glucose, and body weight were measured. Following these measurements, mice were randomly allocated into one of five study groups (initially *n* = 9 per group). Animals in four of these study groups were fed a high-fat, -salt, and -sugar diet (HFSS diet; 42% kcal fat, high glycemic index semi-pure rat diet; SF04-001; Specialty Feeds Perth, WA, Australia) with high-fructose corn syrup (50% glucose and corn) and salt in their drinking water (10% and 0.9%, respectively), to induce MetS. One group of mice was fed a normal chow diet (NCD) with normal drinking water to serve as a control group. These diet regimens were maintained for the remainder of the study until the endpoint. Mice were given ad libitum access to food and water.

### 2.2. Hesperidin Supplementation

Hesperidin (98%, Aktin Chemicals, Inc., Chengdu, China) was administered orally via a method adapted from previous protocols [36]. This was essential to ensure precise dosing and consistent intake among the mice. Administering hesperidin separately from the diet allowed us to control the exact amount each mouse received daily, which can be challenging when mixing it directly into the diet due to potential variations in food consumption. Additionally, incorporating hesperidin into the diet could alter the palatability of the food, potentially affecting the overall food intake and leading to confounding results.

In brief, jellies were prepared weekly, with each hesperidin dose weighed individually for each dose group. The average body weight of mice in the five different experimental groups was used to calculate the hesperidin dose. The jelly mixtures were made using 1300 µL of 8% gelatin stock (Healthy Life Pharmacy, Adelaide, SA, Australia), 450 µL of 2% sucralose, and 150 µL strawberries and cream flavoring (Queen Imitation Flavoring, Australia). Sucralose was used instead of sucrose since it does not stimulate insulin hormone secretion or incretin release and hence has minimal impact on glucose homeostasis [36]. Each jelly contained 250 µL of jelly mix with the appropriate amount of hesperidin powder added (no powder was added to placebo jellies). The jellies were then placed in a molding tray for ~2 h to set and refrigerated overnight in an airtight plastic pocket at 4 °C to ensure they did not dry out. Jellies were kept at 4 °C for up to 7 days, or at −20 °C for up to 3 months.

To allow animals to become accustomed to eating jellies, all mice were first fed placebo jellies for a 2-week training period (9–11 weeks of age). This involved leaving jellies overnight in each mouse cage for familiarization for the first two days. For the next 5 days, mice were moved into separate cages with one littermate and given about 2 h to eat the jellies. Hesperidin supplementation started in week 12. Mice were moved into individual cages daily during jelly feeding to ensure each mouse was eating a full jelly. All mice were fed jellies daily, with each mouse being allocated one of the varying dose groups for the duration of the supplementation period. Mice were given ~1 h to eat the jelly. Any mice that did not eat the full dose were excluded from the study.

### 2.3. Blood Pressure and Body Weight

Systolic blood pressure (BP) was measured non-invasively by tail cuff using the MC4000 Multi-Channel BP Analysis System as described previously (Hatteras Instruments, Cary, NC, USA) [37]. BP was measured fortnightly from baseline (before diet intervention) to week 16 (endpoint). Mice were placed on the BP machine for 40 cycles of 10 measurements, with the first 10 measurements excluded to account for acclimation. After BP measurement, mice were also placed on scales to record their fortnightly body weight.

### 2.4. Glycemic Status

Glycemic status was assessed by conducting fortnightly fasting blood glucose (FBG) measurements throughout the study [38]. Mice were fasted for 6 h, blood was collected via the saphenous vein, and glucose concentrations were measured [38]. A total of 100–200 µL of blood was collected into a heparinized tube and centrifuged for 10 min at 10,000 RPM at 4 °C. Plasma was collected and stored in a separate tube at −80 °C for further analysis. The homeostatic model assessment for insulin resistance (HOMA-IR) was used as an indirect method to quantify insulin sensitivity (calculated by fasting plasma glucose (mg/dL) × plasma insulin (uU/mL)/22.5) [39].

### 2.5. Plasma Analyses

Enzyme-linked immunosorbent assays (ELISAs) were used to quantify plasma DPP-4, 8-OHDG, CRP, and insulin levels. For all analyses, commercially available kits were used (Supplementary Table S1). All samples, standards, and controls were measured in triplicate (DPP-4) or duplicate (8-OHDG, CRP and insulin), and assays were conducted as per the manufacturer’s instructions. Microplates were read on a Multiskan microplate photometer absorbance instrument (Thermo Fisher Scientific, Markham, ON, Canada) at 450 nm to detect the absorbance signal. The intensity of the absorbance signal was proportional to the concentration of the target present in the sample.

### 2.6. Post-Mortem Analyses

Mice were killed via carbon dioxide asphyxiation. Briefly, the heart was exposed with an abdominal incision through the diaphragm, and 0.1 mL of Clexane enoxaparin sodium (400 U/mL, Sanofi, Macquarie Park, NSW, Australia) was injected in the ventricles to minimize clotting when collecting blood from the heart. Blood was collected from the right ventricle using the cardiac puncture method (approximately 0.7–1.0 mL). Whole blood was used to measure HbA1c, CRP, total cholesterol, TGs, and HDL using Cobas discs and run on a Cobas B 101 System (Roche Diagnostics, North Ryde, NSW, Australia).

### 2.7. Measurement of Immune Cell Populations Using Flow Cytometry

Blood was collected from each mouse at the study endpoint and was prepared into single-cell suspensions for flow cytometry analysis as previously described [40]. The blood was centrifuged (Eppendorf South Pacific Pty. Ltd., Macquarie Park, NSW, Australia) for 10 min at 10,000 RPM at 4 °C to separate plasma and other cells. The plasma was separated into a clean tube and snap-frozen for further analysis. The remaining blood was mixed with 10 mL of red blood cell (RBC) lysis buffer (0.15 M NH_4_Cl, 0.01 M KHCO_3_, dH_2_O) and incubated for 5 min. After 5 min, PBS was added, and the cell suspension was centrifuged (Eppendorf 5425 R, NSW, Australia) at 3000× *g* at 4 °C for 5 min. The supernatant was discarded, and RBC lysis was repeated once more. Approximately 1 million cells were loaded onto a 96-well plate (353077; Corning, Mulgrave, VIC, Australia) ready for cell surface marker staining.

Cells were stained with a live/dead viability stain (1:1000 dilution in PBS; Invitrogen, Thermo Fisher, Scoresby, VIC, Australia) for 15 min at room temperature, washed with MACS buffer (0.5% bovine serum albumin and 2 mM EDTA in PBS), and then spun at 3000× *g* at 4 °C for 5 min. The supernatant was discarded, and cells were stained with a cocktail of antibodies against various immune cell markers (Supplementary Table S2). After a 20 min incubation at room temperature, cells were rinsed with MACS buffer and centrifuged at 3000× *g* at 4 °C for 5 min. Subsequently, the liquid above the cell pellet was removed, and the cells were preserved by immersing them in 2% formalin diluted in MACS buffer. The fixed cells were then stored overnight at 4 °C. All samples were analyzed using a CytoFLEX S analyzer (Beckman Coulter, Indianapolis, IN, USA) and CytExpert software version 2.5 (Beckman Coulter, Indianapolis, IN, USA). Immune cell populations were quantified using FlowJo software (BD Biosciences (Franklin Lakes, NJ, USA), version 10.8.1; gating strategy provided in Supplementary Figure S1).

### 2.8. Statistical Analysis

All results are expressed as the mean ± SEM, where n represents the number of animals per group. GraphPad Prism 9.4.1 (GraphPad Software, San Diego, CA, USA) software was used for statistical analysis. Physiological data were analyzed using either a one-way repeated analysis of variance (1-way ANOVA) for endpoint measures or two-way mixed effects ANOVA for timepoint measures. Physiological measures used a Dunnett post hoc test comparing all study groups to the HFSS + placebo group. The level of significance was set at *p* < 0.05 for all statistical analyses.

## 3. Results

### 3.1. Effect of 16-Week HFSS Diet on Physiological Parameters

**Figure 1:**
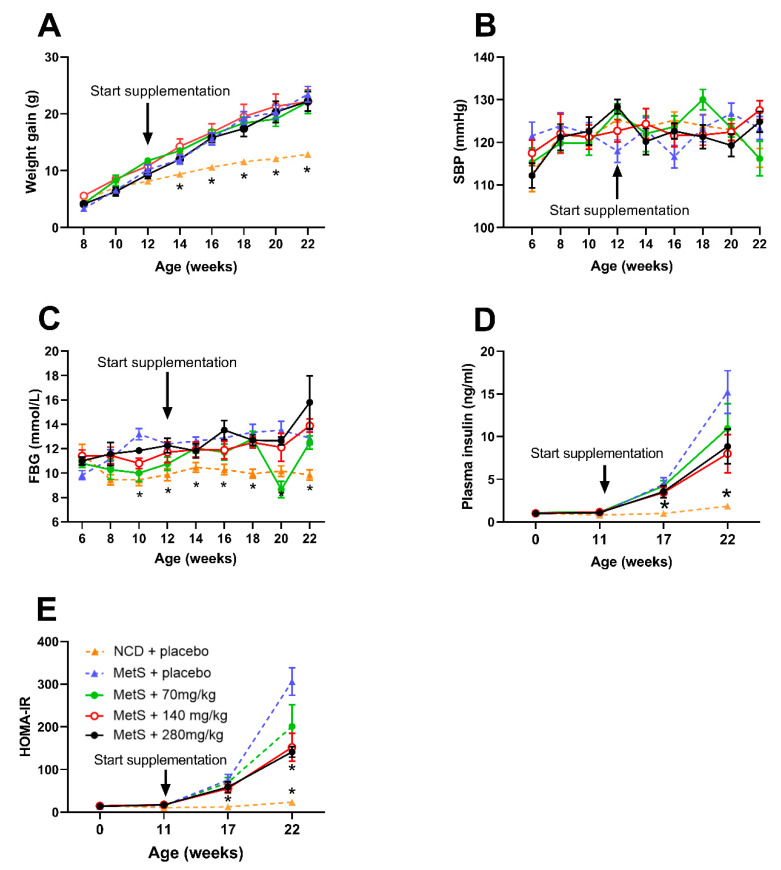
HFSS diet induced increased weight gain and hesperidin’s impact on SBP and weight gain. Weight gain (g; **A**), systolic blood pressure (mmHg; **B**), fasting blood glucose (FBG; mmol/L; **C**), plasma insulin (ng/mL; **D**), and homeostatic model assessment for insulin resistance (HOMA-IR; **E**). Male C57BL6 mice began supplementation at 12 weeks of age (black arrow) with either a placebo (NCD = orange; HFSS = purple) or one of three doses of hesperidin daily (70 mg/kg = green, 140 mg/kg = red, or 280 mg/kg = black). Data are mean ± SEM, *n* = 9 per group. * *p* < 0.05, significantly different to HFSS + placebo group. HFSS: high fat, salt, and sugar; SBP: systolic blood pressure; NCD: normal chow diet (control group).

**Figure 2:**
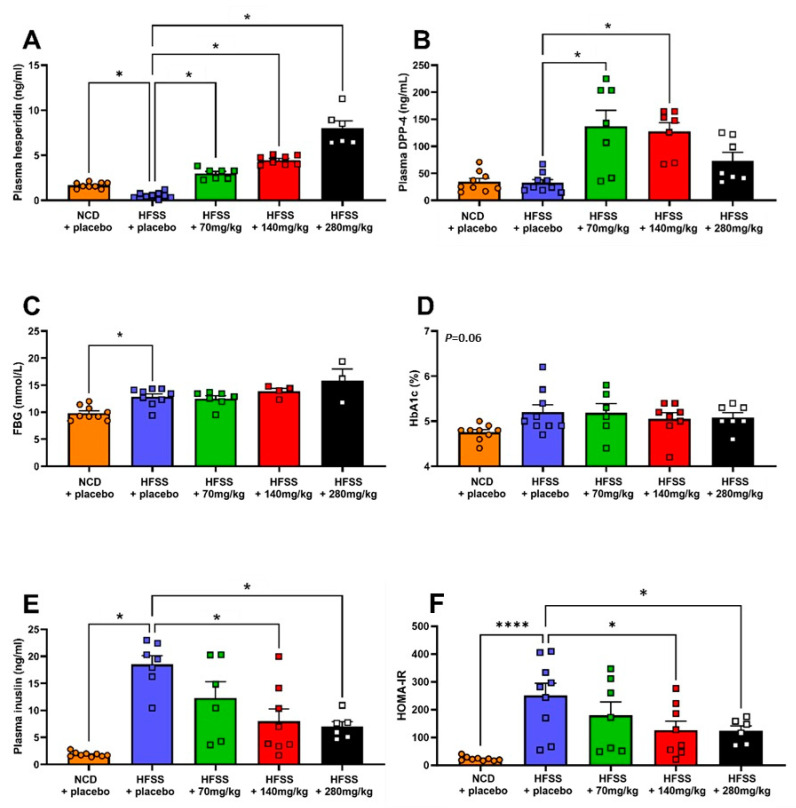
Hesperidin increased DPP-4 levels and reversed insulin resistance. Plasma hesperidin (ng/mL; **A**), plasma dipeptidyl peptidase 4 levels (DPP-4, ng/mL; **B**), glycated hemoglobin (HbA1c; %; **C**), fasting blood glucose (FBG; mmol/L; **D**), plasma insulin (ng/mL; **E**), and homeostatic model assessment for insulin resistance (HOMA-IR; **F**) at endpoint. Male C57BL6 mice were supplemented with either a placebo (NCD = orange; HFSS = purple) or one of three doses of hesperidin daily (70 mg/kg = green, 140 mg/kg = red, or 280 mg/kg = black). Data are mean ± SEM, *n* = 9 per group. * *p* < 0.05, **** *p* < 0.001, significantly different to HFSS + placebo group. HFSS: high fat, salt, and sugar; NCD: normal chow diet (control group).

The HFSS diet induced metabolic disturbances indicative of MetS. Specifically, HFSS + placebo mice (HFSS diet group) gained almost twice as much weight compared to control mice over the 16-week feeding period (*p* < 0.0001; Figure 1A). While there was no effect of the HFSS diet on SBP (Figure 1B), fasting blood glucose and plasma insulin levels were significantly higher in the HFSS diet group by the endpoint compared to the control group (*p* < 0.0001 and *p* < 0.05, respectively; Figure 1C,D). Moreover, at the endpoint, the HOMA-IR index was increased, indicating that the HFSS diet induced insulin resistance (*p* < 0.0004, Figure 1E). At the endpoint, circulating hesperidin levels were significantly lower in the HFSS diet group compared to control group (*p* = 0.03; Figure 2A).

### 3.2. Effect of Hesperidin Supplementation on Physiological Parameters

Throughout the diet regime, hesperidin had no significant effect on weight gain (Figure 1A), SBP (Figure 1B), or fasting blood glucose levels (Figure 1C). However, hesperidin supplementation (140 mg/kg/day and 280 mg/kg/day) significantly lowered insulin levels (*p* < 0.0001 and *p* < 0.005, respectively) and HOMA-IR (*p* < 0.0001 for both parameters, Figure 2D,E) compared to the HFSS diet group. At the study endpoint, there was a significant increase in plasma hesperidin levels in all three hesperidin groups (70 mg/kg/day, 140 mg/kg/day, 280 mg/kg/day) compared to the HFSS diet group (*p* < 0.0001, Figure 2A). Plasma DPP-4 levels were significantly increased in low-dose hesperidin-treated mice (Figure 2B). Post hoc analyses revealed that DPP-4 levels were only significantly raised following the 70 and 140 mg/kg/day hesperidin regimes (*p* < 0.0001 and *p* < 0.0005, respectively). Fasting blood glucose and HbA1c were not affected by hesperidin supplementation (Figure 2C,D). Additionally, HOMA-IR values were significantly lower in the 140 and 280 mg/kg/day hesperidin groups compared with the HFSS diet group (*p* < 0.05 for both parameters, Figure 2E,F).

### 3.3. Effect of HFSS Diet and Hesperidin on Blood Lipids

**Figure 3:**
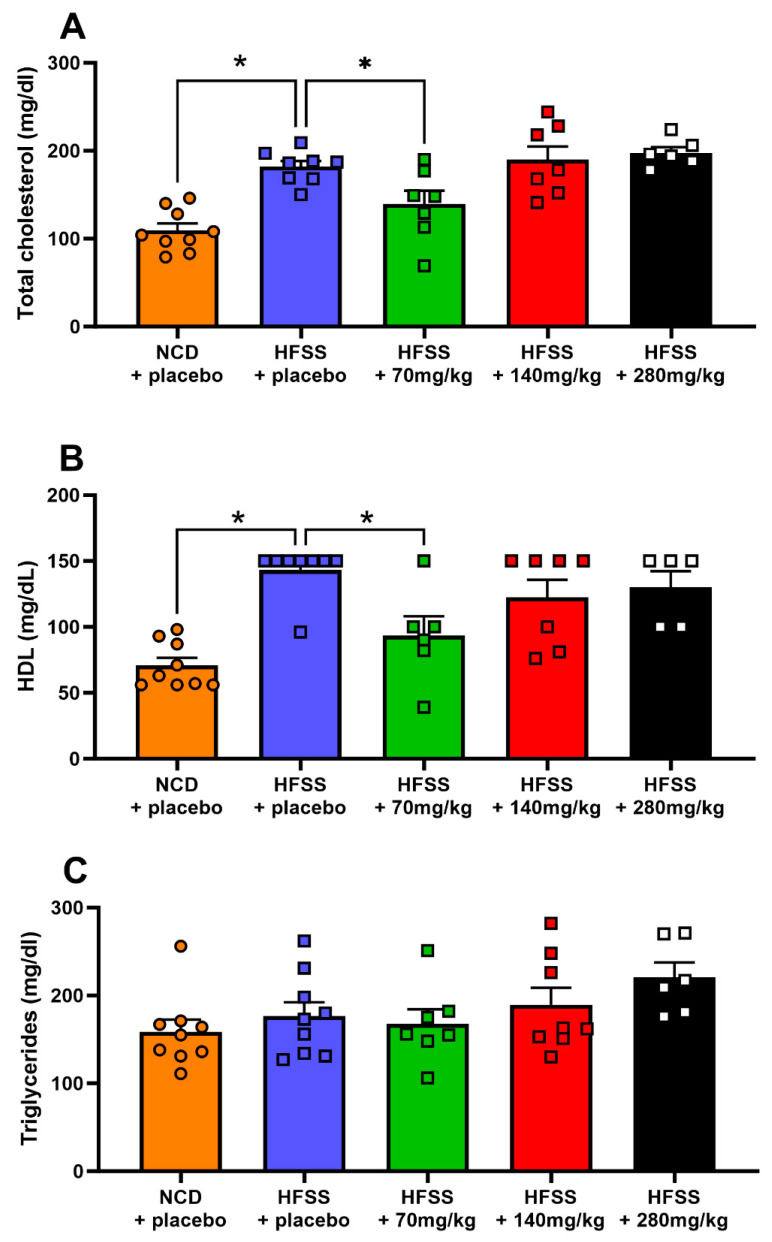
Hesperidin decreased total blood cholesterol and HDL. Total blood cholesterol (mg/dL; **A**), high-density lipoprotein (HDL; mg/dL; **B**), and triglyceride (mg/dL; **C**) levels were measured at study endpoint. Male C57BL6 mice were supplemented with either a placebo (NCD = orange; HFSS = purple) or one of three doses of hesperidin daily (70 mg/kg = green, 140 mg/kg = red, or 280 mg/kg = black). Data are mean ± SEM, *n* = 9 per group. * *p* < 0.05, significantly different to HFSS + placebo group. HFSS: high fat, salt, and sugar; NCD: normal chow diet (control group).

Total blood cholesterol and HDL levels were significantly increased in the HFSS diet group compared to control mice at the endpoint (*p* < 0.0001, Figure 3A,B). Supplementation with 70 mg/kg/day hesperidin significantly decreased total cholesterol and HDL compared to the HFSS diet group (*p* < 0.05 and *p* < 0.005, respectively; Figure 3A,B). The higher doses of hesperidin (140 and 280 mg/kg/day), however, did not significantly alter total cholesterol or HDL levels. Moreover, neither HFSS nor hesperidin significantly affected blood TG levels at the endpoint (Figure 3C).

### 3.4. Effect of HFSS Diet and Hesperidin on Inflammation and Oxidative Stress

**Figure 4:**
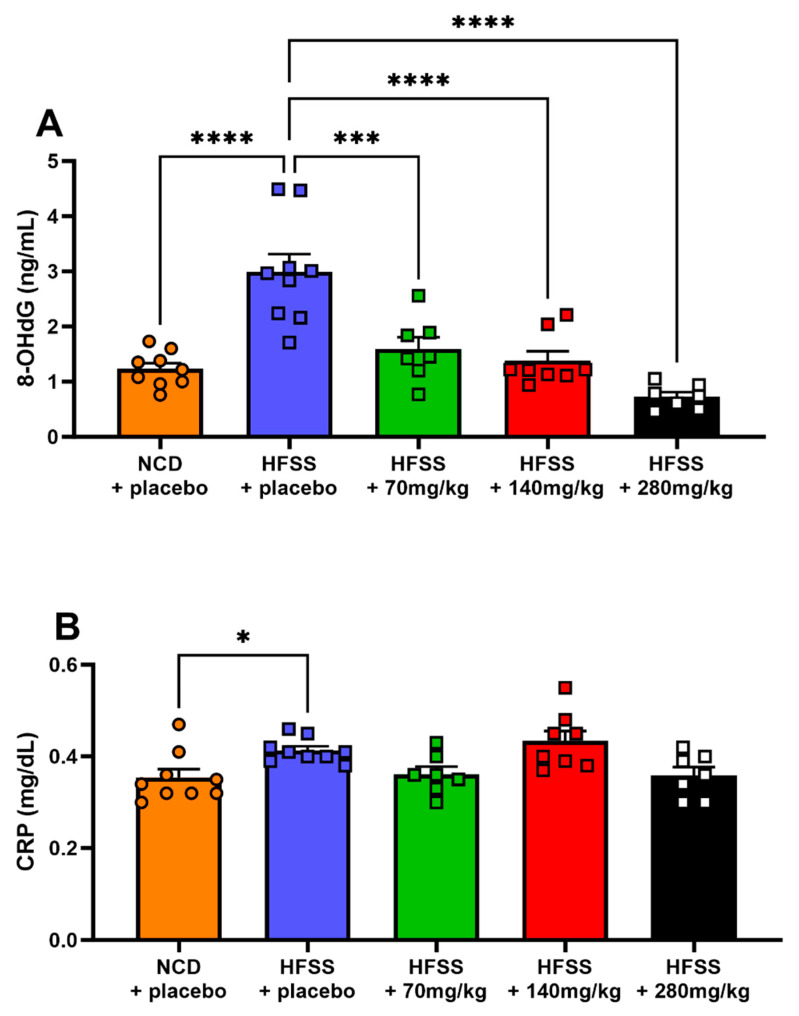
Hesperidin decreased oxidative stress but did not affect inflammation. Plasma levels of 8-hydroxy-2′-deoxy-guanosine (8-OHdG, ng/mL; **A**) and C-reactive protein (CRP, mg/dL; **B**) were measured at study endpoint. Male C57BL6 mice were supplemented with either a placebo (NCD = orange; HFSS = purple) or one of three doses of hesperidin daily (70 mg/kg = green, 140 mg/kg = red, or 280 mg/kg = black). Data are mean ± SEM, *n* = 9 per group. * *p* < 0.05, *** *p* < 0.01, **** *p* < 0.001, significantly different to HFSS + placebo group. HFSS: high fat, salt, and sugar; NCD: normal chow diet (control group).

At the study endpoint, oxidative stress, as quantified by 8-OHdG, was elevated in the HFSS diet mice compared to the control mice (*p* < 0.001; Figure 4A). Importantly, plasma 8-OHdG levels were significantly reduced in all three study groups of mice receiving hesperidin supplementation (*p* < 0.005; Figure 4A). Inflammatory status, assessed using the proinflammatory marker CRP, was significantly elevated in HFSS diet mice compared to control mice (*p* < 0.05, Figure 4B). Hesperidin supplementation did not significantly alter plasma CRP levels (Figure 4B).

### 3.5. Effect of HFSS Diet and Hesperidin on Circulating Immune Cells

**Figure 5:**
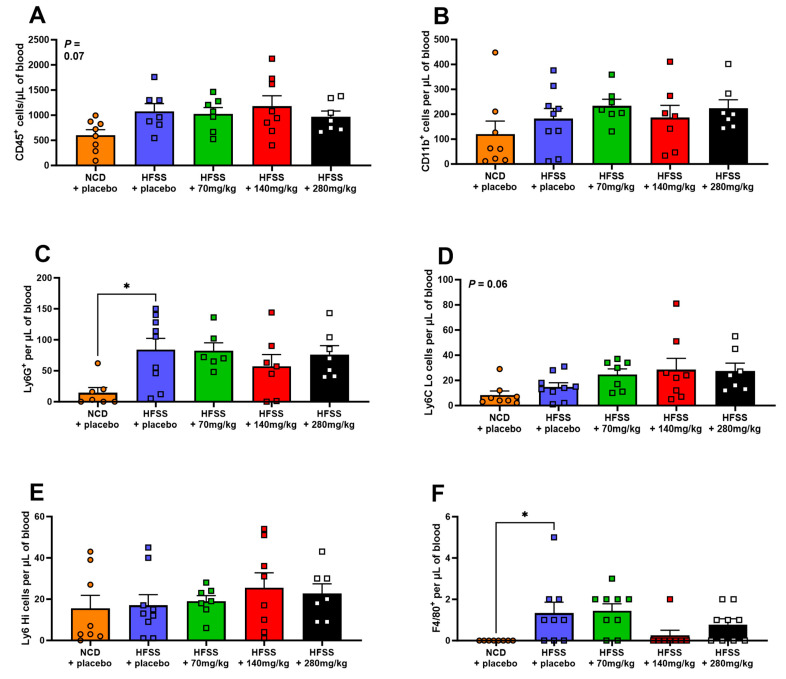
Hesperidin did not significantly affect numbers of myeloid-derived immune cells in blood. Leukocytes (CD45^+^; **A**), myeloid-derived cells (CD11b^+^; **B**), neutrophils (Ly6G^+^; **C**), patrolling monocytes (Ly6C^Lo^; **D**), proinflammatory monocytes (Ly6C^Hi^; **E**), and macrophages (F480^+^; **F**) were detected using flow cytometry at study endpoint. Male C57BL6 mice were supplemented with either a placebo (NCD = orange; HFSS = purple) or one of three doses of hesperidin daily (70 mg/kg = green, 140 mg/kg = red, or 280 mg/kg = black). Data are mean ± SEM, *n* = 9 per group. * *p* < 0.05, significantly different to HFSS + placebo group. HFSS: high fat, salt, and sugar; NCD: normal chow diet (control group).

**Figure 6:**
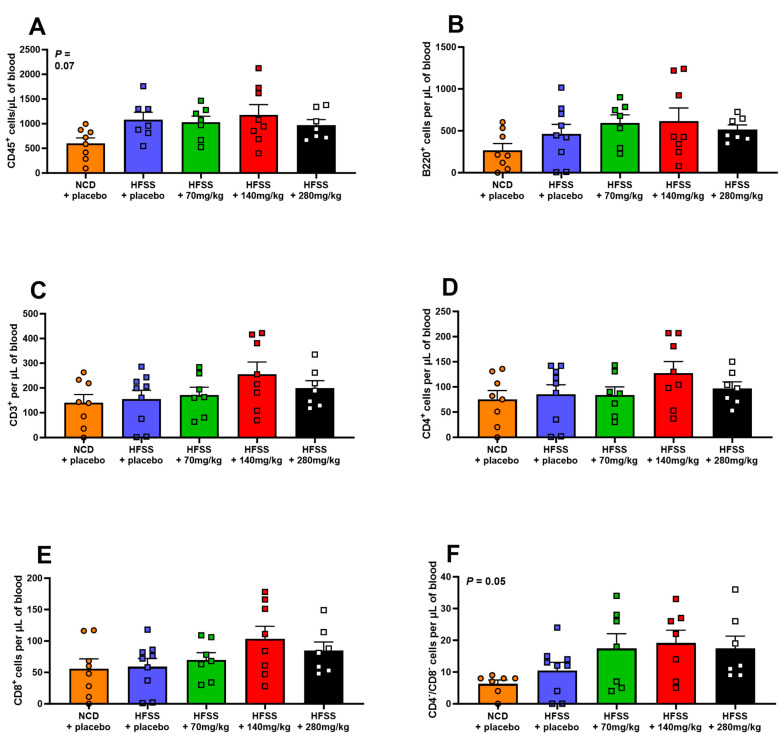
Hesperidin did not significantly affect blood lymphocyte numbers in the blood. Leukocytes (CD45^+^; **A**), B cells (B220^+^; **B**), T cells (CD3^+^; **C**), T regulatory cells (CD4^+^; **D**), cytotoxic T cells (CD8^+^; **E**), and double-negative T cells (CD4^−^/CD8^−^; **F**) were detected using flow cytometry at study endpoint. Male C57BL6 mice were supplemented with either a placebo (NCD = orange; HFSS = purple) or one of three doses of hesperidin daily (70 mg/kg = green, 140 mg/kg = red, or 280 mg/kg = black). Data are mean ± SEM, *n* = 9 per group. HFSS: high fat, salt, and sugar; NCD: normal chow diet (control group).

The HFSS diet did not affect the number of total circulating leukocytes (CD45^+^), total myeloid-derived cells (CD11b^+^), Ly6C^hi^ monocytes, or any of the analyzed lymphocyte populations (Figure 5A–D and Figure 6). In contrast, neutrophil (Ly6G^+^) and macrophage (F4/80^+^) numbers significantly increased in mice fed the HFSS diet alone compared to NCD (*p* < 0.01 and *p* = 0.03, Figure 5C,F). Hesperidin supplementation in HFSS-fed mice did not significantly affect leukocyte subpopulations, but there was a notable trend (*p* = 0.07 and 0.06), where higher doses appeared to reduce elevated macrophage levels (F480^+^) (Figure 5 and Figure 6).

## 4. Discussion

This study is one of only a few to evaluate the dose-specific physiological responses to hesperidin and the only one to test this in the setting of MetS. In the present study, 12 weeks of hesperidin supplementation prevented hyperinsulinemia and hypercholesterolemia as associated with long-term ingestion of a HFSS diet. It also reduced HFSS-induced oxidative stress and tended to prevent the increase of circulating macrophages. Interestingly, despite hesperidin being previously known for its DPP-4 inhibitory properties, an unexpected outcome was observed, where plasma DPP-4 levels were actually elevated in mice subjected to a HFSS diet (diet-induced MetS). This paradoxical increase can be understood in light of recent insights suggesting that prolonged inhibition of DPP4 not only elevates plasma levels of soluble DPP4 (sDPP4) but also stimulates sDPP4 expression in lymphocyte-rich organs in mice [41]. Thus, the findings from this study not only underscore hesperidin’s beneficial impact on various metabolic disruptions linked to MetS but also illuminate the complex interplay between hesperidin supplementation and DPP-4 dynamics, offering a nuanced perspective on its pharmacological effects.

In the present study, plasma hesperidin concentrations increased with increasing hesperidin supplementation dosage, demonstrating that oral hesperidin was absorbed into the circulation in a dose-specific manner. Interestingly, while plasma hesperidin levels were negligible in mice fed a HFSS diet alone, there were detectable levels in mice fed a normal chow diet (NCD). This was an intriguing finding, as neither of these groups was supplemented with hesperidin. Further investigation of the scientific literature revealed that soybeans, a predominant ingredient in the NCD, contain traces of hesperidin [42]. This suggests that hesperidin levels may be lower in individuals consuming an unhealthy/western diet low in plant-based foods, likely due to reduced polyphenol intake. The presence of hesperidin in the control group’s diet raises important considerations. The similar plasma hesperidin levels in the NCD + placebo group and the group treated with 70 mg/kg of hesperidin indicate that a diet rich in plant-based foods might be sufficient to confer some of the beneficial effects against MetS. This underscores the potential of a balanced diet in mitigating metabolic disturbances.

While we observed dose-dependent increases in plasma hesperidin concentrations, corresponding effects on various metabolic parameters were not consistently dose-dependent. This variability can be attributed to several factors. Different metabolic parameters may have varying sensitivities to hesperidin, with some responding more readily to changes in hesperidin concentration than others. The mechanisms of hesperidin’s effects are complex and may involve multiple pathways, leading to non-linear dose responses. Biological variability, including differences in metabolism, absorption, and tissue distribution, also contributes to these inconsistencies. Additionally, threshold effects may occur, where certain doses of hesperidin are sufficient to trigger a response, and further increases do not enhance the effect. The study duration may also play a role, as some parameters might require longer treatment periods to exhibit dose-dependent responses.

In the current study, the three doses of hesperidin were chosen based on a previous study that demonstrated significant increases in total antioxidant capacity and reductions in inflammation with hesperidin supplementation at 140 mg/kg/day in mice [43]. To provide context for these doses in a human population, it is important to translate these amounts into equivalent dietary intake. The highest dose used in our study, 280 mg/kg/day, far exceeds the typical dietary intake and would be impractical to achieve through normal consumption. For instance, a medium-sized orange contains approximately 20–60 mg of hesperidin [23]. Translating the 140 mg/kg/day dose for humans, considering an average human weight of 70 kg, would equate to approximately 9.8 g of hesperidin daily. This is equivalent to consuming about 196–490 oranges per day, which underscores the impracticality of achieving these levels through diet alone. These findings highlight the potential need for hesperidin supplementation to achieve the therapeutic benefits observed in the study.

To date, most studies exploring the therapeutic potential of hesperidin have used short-term (up to 8 weeks) treatment protocols of genetically or drug-induced T2DM rodent models. A previous study showed STZ-treated Wistar rats fed a high-fat diet had increased FBG levels. A 100 mg/kg daily supplementation of hesperidin for 4 weeks reduced FBG levels by 44.7% [44]. Another study in *db*/*db* (leptin receptor deficient) mice showed that treatment with 200 mg/kg of hesperidin for 5 weeks had a marked glucose-lowering effect of ~35% [45]. By contrast, in the current study, we observed no significant effects of hesperidin treatment on FBG levels in HFSS-fed mice. This is consistent with a previous study where 0.3 g/L hesperidin supplementation for 90 days in the drinking water of hyperglycemic Wistar rats (administered high-sucrose drinking water) also had no effect on FBG levels [46]. A recent systematic review/meta-analysis of the clinical trial literature identified six trials with 318 participants. The results showed that hesperidin had no significant effect on serum fasting blood glucose (weighted mean difference [WMD] = −1.10 mg/dL, 95% CI: −3.79, 1.57) [47]. Conversely, a clinical trial involving 49 MetS patients found that 500 mg of hesperidin supplementation, twice daily for 12 weeks, significantly lowered FBG levels by ~54% [33].

There was a significant difference in FBG levels observed at week 10 between the NCD + placebo group and the HFSS + placebo group despite the lack of alterations in HOMA-IR. This can be attributed to the dietary differences between the groups. By week 10, the mice in the HFSS + placebo group had been on the high-fat, high-salt, high-sugar (HFSS) diet for four weeks, which is known to induce early metabolic changes. These early changes can occur before significant insulin resistance is detectable through HOMA-IR. The diet’s high sugar content can rapidly impact blood glucose levels independently of insulin sensitivity.

Although the current study did not show significant effects on FBG levels overall, the decrease observed at week 20 in the HFSS + 70 mg/kg group is noteworthy. Moreover, hesperidin reduced diet-induced hyperinsulinemia. Both 140 and 280 mg/kg/day hesperidin supplementation significantly decreased insulin levels in mice with the HFSS diet (diet-induced MetS) by the study endpoint. In addition, HOMA-IR was significantly reduced in these treatment groups, providing evidence that the higher doses of hesperidin supplementation reduced insulin resistance. The hypothesis that reduced circulating insulin levels may be due to increased glucose absorption into organs such as the liver, skeletal muscle, and adipose tissue is based on previous findings [32,48,49]. Hesperidin treatment has been reported to increase GLUT2/4 receptor expression and activate the AKT pathway in some of these organs [32,48,49]. Moreover, dietary supplementation of 200 mg/kg hesperidin for 5 weeks in a *db*/*db* T2DM mouse model caused upregulation of hepatic glucokinase (a sensitive measurement of glucose uptake in the liver) [50]. These findings suggest that activation of AKT and subsequent upregulation of GLUT receptors may be a key mechanism by which hesperidin helps to control hyperinsulinemia. However, it is important to note that the mice in our study continued receiving the HFSS diet throughout the study period, which likely contributed to the persistently high FBG levels across all hesperidin-treated groups. This dietary factor may have masked potential improvements in blood glucose levels. Further studies are required to determine whether hesperidin affects GLUT2/4 and AKT in the liver of mouse model with MetS, which could explain the prevention of insulin resistance despite the lack of change in blood glucose levels. This area remains an important direction for future research [47].

Our previous molecular docking study suggested that hesperidin can bind to an active site of the DPP-4 enzyme—the same site that is targeted by clinically relevant gliptins [34]. Moreover, isolated enzyme studies indicated that hesperidin inhibits DPP-4 activity with similar efficacy (albeit lower potency) to gliptins. Hence, it was somewhat surprising in the present study that the two lower doses of hesperidin (70 and 140 mg/kg/day) significantly increased DPP-4 enzyme levels in the circulation of HFSS-fed mice. The increased DPP-4 levels in these groups was inversely correlated with the plasma insulin levels, as per previous studies [51]. However, the highest hesperidin dose (280 mg/kg/day) did not significantly increase DPP-4 levels. This finding could be explained by a potential biphasic dose–response curve associated with phytochemicals, wherein varying doses can lead to diametrically opposed biological effects [25,52]. However, further study is needed to investigate the mechanism behind these observations and to delineate the optimal dose ranges that would maximize therapeutic efficacy while minimizing adverse effects.

The interaction between hesperidin and glucose metabolism is complex and requires a deep understanding of how different doses affect the body. At lower doses, hesperidin can increase the activity of the DPP-4 enzyme, which is inversely related to plasma insulin levels. This suggests that hesperidin can affect glucose metabolism by influencing this enzyme. However, this effect changes at higher doses, where hesperidin might activate other biological responses that neutralize its effect on DPP-4 or reach a limit after which more hesperidin does not enhance enzyme activity further [53]. This complexity is even more evident when considering that, at higher doses, hesperidin could lower insulin levels through different mechanisms, such as affecting GLUT receptors and the AKT pathway, activating AMPK, altering Sirt1, or impacting leptin levels. This indicates a dose-dependent variation in how hesperidin works. Additionally, hesperidin’s role in reducing inflammation and oxidative stress in conditions like respiratory diseases, through pathways such as NF-kB, iNOS, and COX-2, suggests it might also affect insulin levels by pathways other than DPP-4 inhibition [54]. This highlights the intricate workings of biological systems and the importance of carefully determining the right dose for therapeutic use. More research is needed to fully understand the various ways hesperidin influences glucose and insulin metabolism at different doses.

In this study, long-term consumption of the HFSS diet led to increased circulating levels of the oxidative stress marker 8-OHdG, highlighting the diet’s role in promoting oxidative stress [4], which is further exacerbated by insulin resistance, hyperglycemia, obesity, and dyslipidemia [4]. This oxidative stress was associated with a chronic low-grade inflammatory response, evidenced by elevated plasma levels of CRP and increased circulating neutrophils and macrophages, making the HFSS mice a robust model for the low-grade inflammation observed in MetS [11]. Conversely, hesperidin supplementation decreased 8-OHdG levels in a dose-dependent manner, reflecting its antioxidant capabilities through scavenging free radicals and mitigating oxidative stress [30], a key contributor to the pathogenesis of MetS [55]. Despite hesperidin’s antioxidant properties and its potential to inhibit oxidative stress-induced inflammation, a fundamental component of MetS, it did not significantly affect CRP levels. This finding contrasts with previous research indicating hesperidin’s effectiveness in reducing circulating levels of CRP, suggesting a complex interaction between hesperidin supplementation and inflammatory markers in the context of MetS [56].

The observed cholesterol levels in the HFSS + placebo group indeed suggest hypercholesterolemia, aligning with other reports that HFSS diets significantly raise cholesterol levels in rodents, pushing them beyond the normal range. The absence of a clear dose–response relationship in our observations might be due to the specific interaction between hesperidin and the cholesterol biosynthesis pathway. While the HFSS diet elevated total cholesterol and HDL levels, supplementation with a low dose of hesperidin (70 mg/kg/day) effectively mitigated these increases. This observation aligns with the mechanism of action of statins, the primary treatment for high cholesterol and dyslipidemia, [57], which function by inhibiting the hydroxymethylglutaryl-CoA (HMG-CoA) reductase enzyme essential for cholesterol production. Recent studies and our findings suggest that hesperidin may also inhibit HMG-CoA reductase, indicating a threshold effect where low doses are sufficient to significantly impact cholesterol biosynthesis [58]. Additionally, the consistency of hesperidin’s effect on not altering circulating TG levels with previous research underlines the specificity of its action on cholesterol metabolism, possibly explaining why increasing the dose does not enhance the lipid-lowering effect [59].

In the present study, we evaluated specific immune cell subtypes, including total leukocytes (CD45+), myeloid-derived cells (CD11b+), Ly6Chi monocytes, neutrophils (Ly6G+), and macrophages (F4/80+), due to their known involvement in inflammation and metabolic syndrome. These cell types play crucial roles in systemic and tissue-specific inflammatory responses, which are key features of HFSS diet-induced metabolic disturbances. HFSS significantly increased circulating neutrophils and macrophages [60,61]. This correlates with previous findings in humans, where neutrophils were increased in the setting of obesity [60]. Interestingly, HFSS did not affect any of the circulating lymphocyte populations. This contrasts with the current paradigm, whereby circulating pro-inflammatory T cells typically increase in the setting of obesity [61]. Notably, this is the first study to investigate immune cells in MetS in the context of hesperidin treatment. While previous research has underscored the anti-inflammatory effects of hesperidin in T2DM, our investigation revealed no substantial impact on circulating immune cells. However, it is noteworthy that we observed a trend towards a reduction in macrophage levels [30]. Additionally, the potential of hesperidin to attenuate leukocyte activation cannot be discounted, suggesting that its immunomodulatory role warrants further exploration.

In this study, we exclusively utilized male mice as our experimental subjects. This choice was driven by specific objectives and considerations pertinent to the scope and focus of our research. However, it is important to acknowledge the potential implications of not including female mice, which may introduce limitations related to the generalizability of our findings across genders, as there could be sex-specific physiological or behavioral responses that were not accounted for. Despite this, the selection of male mice was deemed necessary to streamline the experimental design and to address the study aims with greater clarity and specificity. Future research could benefit from incorporating both genders to explore any sex-dependent variations in response, thereby providing a more comprehensive understanding of the phenomena under investigation.

Our study has several limitations that should be acknowledged. Firstly, while we measured the amount of DPP-4 enzyme, we did not assess its enzymatic activity. Future studies should include direct measurements of DPP-4 activity for a more comprehensive analysis. Secondly, we did not measure incretin hormones such as GLP-1 and GIP, which are crucial for insulin secretion and glucose homeostasis. Including these measurements would provide deeper insights into hesperidin’s mechanism of action. We also did not perform glucose and insulin tolerance tests, which are essential for assessing glucose and insulin metabolism dynamics. These tests would help better characterize hesperidin’s effects on glucose tolerance and insulin sensitivity.

Moreover, the persistent high-fat, high-salt, high-sugar (HFSS) diet throughout the study may have contributed to the persistently high fasting blood glucose levels in all hesperidin-treated groups, potentially masking some benefits of hesperidin on blood glucose levels. In addition, the NCD diet contained a trace amount of hesperidin, which could influence the baseline metabolic state of the control mice. Also, while we observed dose-dependent increases in blood hesperidin levels, we did not assess its bioavailability. Future studies will include a detailed bioavailability analysis to better understand hesperidin’s absorption and utilization.

## 5. Conclusions

Overall, this study investigated the effects of hesperidin supplementation on glycemic control, inflammation, and oxidative status in mice with an HFSS diet (diet-induced MetS). Overall, this study indicates that long-term 12-week hesperidin supplementation decreased hyperinsulinemia and oxidative stress but had no effect on systemic inflammation in mice with MetS. These effects of hesperidin were associated with a significant rise in DPP-4 activity in the lower-dose hesperidin-treated groups. Total cholesterol was also decreased with the supplementation of low-dose hesperidin (70 mg/kg/day). Ultimately, hesperidin supplementation provided multiple beneficial outcomes in the setting of MetS and may have potential as a stand-alone or adjunct therapy in MetS. Overall, the highest tested dose, 280 mg/kg/day, demonstrated the most beneficial outcomes in terms of reduced insulin resistance and reducing oxidative stress.

## Data Availability

Data are available to view from the authors upon request.

## Supplementary Materials

The following supporting information can be downloaded at: https://www.mdpi.com/article/10.3390/biom14060637/s1, Figure S1: Flow cytometry gating strategy; Table S1: Details of ELISA experiments for plasma analyses; Table S2: Antibody panel used for flow cytometry.
