# Supplementary material for: Reduced Insulin Resistance and Oxidative Stress in a Mouse Model of Metabolic Syndrome following Twelve Weeks of Citrus Bioflavonoid Hesperidin Supplementation: A Dose–Response Study
Source: Biomolecules. 2024 May 29;14(6):637. doi: 10.3390/biom14060637 (PMC11201492; doi:10.3390/biom14060637)
Supplement: Supplementary file 1 [file biomolecules-14-00637-s001.zip › biomolecules-2959708-supplementary.pdf]

Reduced insulin resistance and oxidative stress in a mouse model of metabolic syndrome following twelve weeks of citrus bioflavonoid hesperidin supplementation.

Abdulsatar Jamal[1], Holly Brettle[1], Dina A Jamil[1,2], Vivian Tran[1], Henry Diep[1], Alexander Bobik,[1,3] Chris van der Poel[1,2], Antony Vinh[1], Grant R Drummond[1], Colleen J Thomas[1], Maria Jelinic[1*] and Hayder A Al-Aubaidy[1,2*].

[1]Centre for Cardiovascular Biology and Disease Research & Department of Microbiology, Anatomy, Physiology & Pharmacology, La Trobe University, Bundoora, VIC, Australia 3086.
[2]NewMed Education Australia, Hamilton, QLD, Australia 4007.
[3]Baker Heart and Diabetes Research Institute, Melbourne, Victoria, Australia.
[4]Australian Institute for Musculoskeletal Science, Melbourne, Victoria, Australia
*Joint senior & corresponding author

Running title (5 words): Hesperidin supplementation benefits metabolic syndrome.

Co-corresponding authors:
A/Prof Hayder A Al-Aubaidy, La Trobe University,
Cnr Plenty Road and Kingsbury Drive, Bundoora, Victoria 3086, Australia.
Phone: +61 3 9479 8728
Email: H.Alaubaidy@latrobe.edu.au

Dr Maria Jelinic, La Trobe University,
Cnr Plenty Road and Kingsbury Drive, Bundoora, Victoria 3086, Australia.
Phone: +61 3 9479 3631
E-mail: m.jelinic@latrobe.edu.au

Declarations of interest: none

Suppl Table S1. Details of ELISA experiments for plasma analyses.

Target	Sample dilution	Product number	Detection range	Manufacturer
**DPP-4 (CD26 adeno-sine deaminase)**	1:50	ab264630	93.75 - 6000 pg/ml	Abcam, Melbourne, Australia
**8-OHDG**	1:20	ab285254	1.563 - 100 ng/ml	Abcam, Melbourne, Australia
**Insulin**	None	80-INSMSU-E01	0.18 - 6.9 ng/ml	ALPCO, New Hampshire, USA

DPP-4: Dipeptidyl Peptidase – 4; 8-OHDG: 8-Hydroxy Deoxy Guanosine

Suppl Table S2. Antibody panel used for flow cytometry.

Antigen	Clone	Target cell	Fluorophore	Working concentration
**CD45**	30-F11	Leukocytes	A700	0.5 mg/ml
**CD3**	145-2C11	T cells	APC	0.2 mg/ml
**CD11b**	M1/70	Myeloid cells	BV421	0.2 mg/ml
**CD4**	RM4-5	T Helper cells	BV605	0.2 mg/ml
**CD8**	53.6.7	Cytotoxic T cells	PerCP-Cy5.5	0.2 mg/ml
**F4/80**	BM8	Macrophages	APC-Cy7	0.2 mg/ml
**Ly6G**	1A8	Neutrophils	PE-Cy7	0.2 mg/ml
**Ly6C**	HK1.4	Monocytes	FITC	0.5 mg/ml
**B220**	RA-6B2	B cells	PE	0.2 mg/ml
**CD206**	C068C2	M2-like Macs	PE-Dazzle594	0.2 mg/ml

All antibodies were purchased from Biolegend, San Diego, California, USA.

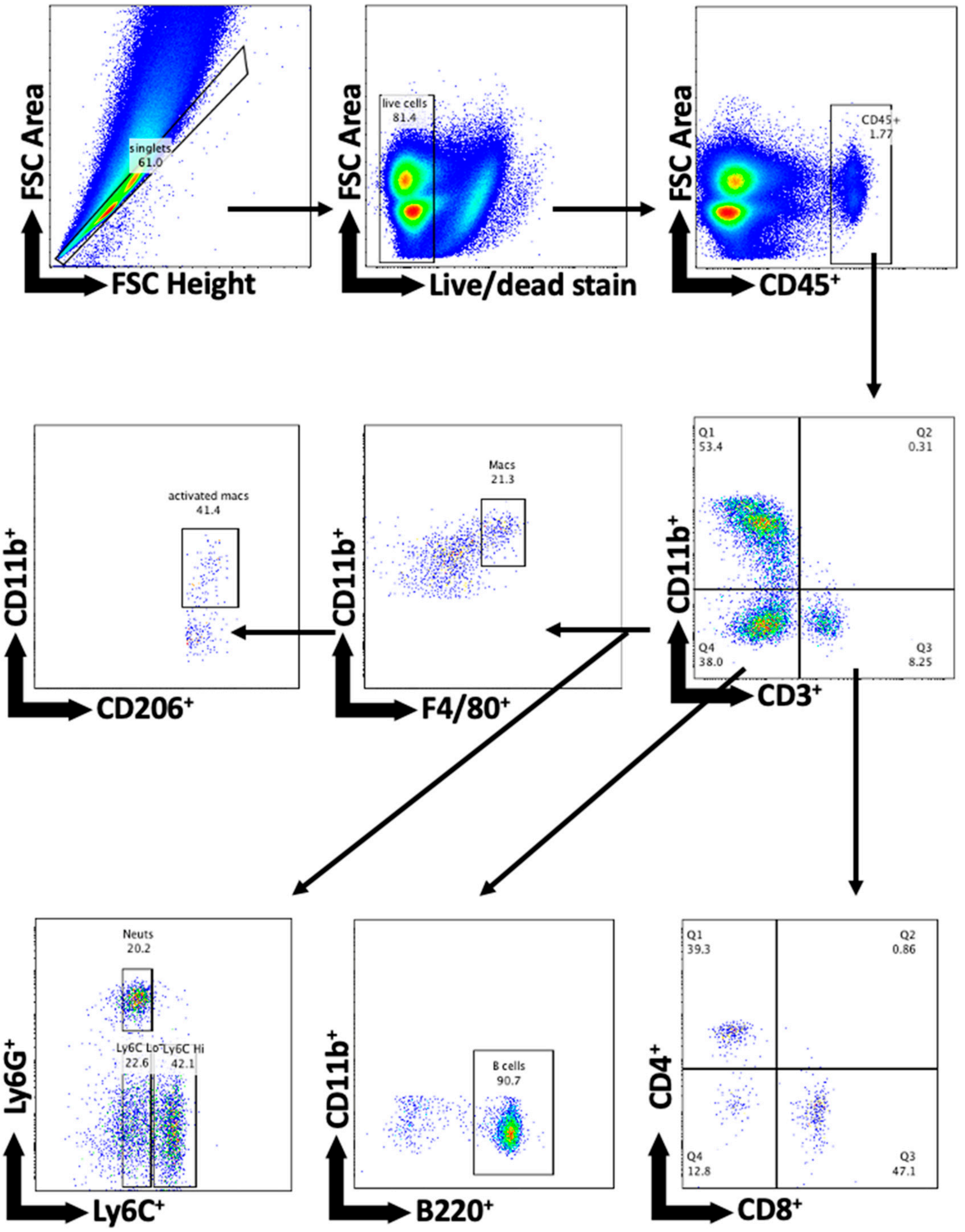

Suppl Figure S1. Flow cytometry gating strategy. Singlets were isolated using forward scatter (FSC) height and FSC area to eliminate doublets or higher. Singlets were gated against live/dead stain to identify live cells, which were then plotted against CD45. $CD45^+$ cells (leukocytes) were gated against $CD11b^+$ and $CD3^+$. Myeloid-derived cells ($CD11b^+/CD3^-$) were plotted against F4/80, Ly6G and Ly6C to identify macrophages, neutrophils and monocytes, respectively. $B220^+/CD11b^+/CD3^-$ cells were classified as B cells. T cells ($CD11b^+/CD3^-$) were further characterised against CD4 and CD8.
